# Biomechanical Modeling of Human Skin Tissue Surrogates

**DOI:** 10.3390/biomimetics3030018

**Published:** 2018-07-23

**Authors:** Arnab Chanda

**Affiliations:** 1 Department of Bioengineering, University of Pittsburgh, Pittsburgh, PA 15213, USA; arc165@pitt.edu; Tel.: +1-205-887-5642; 2 Department of Aerospace Engineering and Mechanics, University of Alabama, Tuscaloosa, AL 35401, USA

**Keywords:** skin, tissue, surrogate, anisotropy, hyperelastic

## Abstract

Surrogates, which precisely simulate nonlinear mechanical properties of the human skin at different body sites, would be indispensable for biomechanical testing applications, such as estimating the accurate load response of skin implants and prosthetics to study the biomechanics of static and dynamic loading conditions on the skin, dermatological and sports injuries, and estimating the dynamic load response of lethal and nonlethal ballistics. To date, human skin surrogates have been developed mainly with materials, such as gelatin and polydimethylsiloxane (PDMS), based on assumption of simplified mechanical properties, such as an average elastic modulus (estimated through indentation tests), and Poisson’s ratio. In addition, pigskin and cowhides, which have widely varying mechanical properties, have been used to simulate human skin. In the current work, a novel elastomer-based material system is developed, which precisely mimics the nonlinear stress–stretch behavior, elastic modulus at high and low strains, and fracture strengths of the natural human skin at different body sites. The manufacturing and fabrication process of these skin surrogates are discussed, and mechanical testing results are presented.

## 1. Introduction

**Figure 1:**
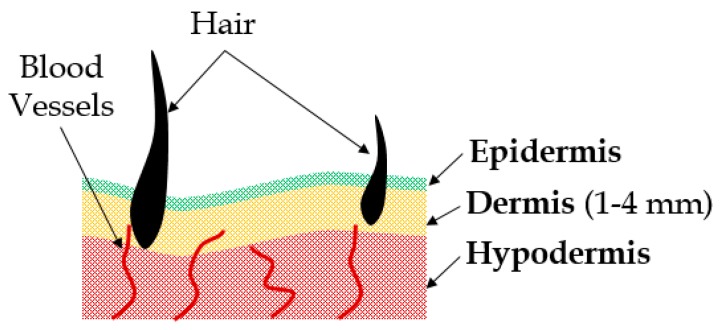
Skin layers and components.

The skin is the outermost protective tissue on the human body, which is composed of three layers, namely the epidermis (the top layer), the dermis (the middle layer) and hypodermis (the bottom layer), with an approximate thickness of 3 mm [1] (see Figure 1). For any external loads applied on the body, throughout the lifespan of an individual, the skin is the first point of contact. The skin is also the first barrier against any physical injury. In the last two decades, there were several attempts at developing artificial skin replacements for severe burn injuries [2]. The focus was on recreating the biocompatibility and mechanical properties of the natural skin. The mechanical properties of human skin [3,4,5,6,7] were studied extensively through uniaxial [8,9,10,11], biaxial [12,13,14,15,16], multiaxial [17] and indentation tests [18,19,20,21], under both static and dynamic loading [22,23,24,25] conditions, as well as using a plethora of imaging techniques [19,26,27]. It was observed that skin exhibits nonlinear material behavior and stiffening with the increase in the applied load from the mechanical point of view. Several phenomenological material models of the skin [28] were developed in the past for burned skin replacements [2] and surgical needle insertion simulations [29], to study skin optical properties [30,31], perfusion [32], sports injuries [33,34], and the penetration resistance of ballistics [35,36,37,38]. However, to date, a key gap in the literature exists with respect to the simulation of the realistic nonlinear material properties of the human skin, which would be indispensable for biomechanical testing applications, such as estimating the load response of cosmetic implants and the biomechanical study of skin injuries. In addition, an ongoing goal in the ballistic community has recently been reported to develop less-lethal munitions [39], aimed at deterring individuals without causing fatal injuries. A surrogate with realistic mechanical properties of the human skin is imperative to test the load response of such munitions prior to their deployment in the field.

In the current work, based on the concept of silicone breast implants [40], and extensive mechanical testing data on the human skin [8,10,13,35,41,42,43,44,45,46,47,48], surrogates have been developed to simulate the mechanical properties of the skin at different sites on the human body. The mechanical testing, manufacturing and fabrication techniques are presented in detail. In addition, the mechanical characterization of the developed surrogates using hyperelastic material models is discussed. The results of the mechanical tests conducted on the human skin surrogates are discussed along with the comparison of the mechanical properties of the surrogates with the natural skin. Additionally, some immediate and future application areas of the developed novel human skin surrogates are presented.

## 2. Materials and Methods

### 2.1. Fabrication of Human Skin Tissue Surrogates

**Figure 2:**
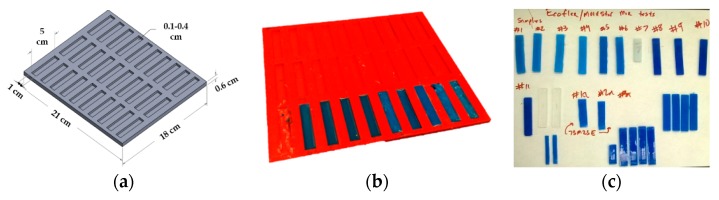
Skin surrogate fabrication. (**a**) Mold design for test coupons in Solidworks; (**b**) three-dimensional (3D) printing of the mold for test coupon preparation; and (**c**) specimens with different sizes and color variations (implying different mix ratios).

Elastomer materials were characterized using the Shore durometer hardness scale, defined as per the American Society for Testing and Materials (ASTM) D2240 testing standard [49]. An extremely soft two-part elastomer material with a Shore hardness of 00-10 (Ecoflex 0010, Smooth-On, Inc., Macungie, PA, USA) was combined with a stiffer two-part elastomer material with a Shore hardness of 30A (Mold Star 30, Smooth-On, Inc.) to fabricate the human skin surrogates [44,45,50]. A mold was designed (Figure 2a) in Solidworks computer-aided design (CAD) software (Dassault Systems, Waltham, MA, USA) to produce 30 coupons with the dimension of 5 cm in length, 1 cm in width and 3 mm in depth. The overall dimension of the mold was 21 cm × 18 cm × 1 cm. This mold design was exported as a stereolithographic (STL) file and sent for three-dimensional (3D) printing at the University of Alabama 3D printing lab. The 3D printer used was a Dimension SST 1200es (Stratasys Inc., Eden Prairie, MN, USA), and the material used for fabricating the mold was acrylonitrile butadiene styrene (ABS), a very popular material used in the 3D printing industry, which provides a good surface finish and high strength. The 3D printed mold is shown in Figure 2b.

To fabricate the human skin surrogates, 55 test coupons with a similar dimension (49–50 mm length, 9–11 mm width, and 2.8–3.1 mm thickness) were prepared with different weight percentages of the four-part elastomer. Table 1 summarizes the 23 specific composition batches fabricated for uniaxial testing in this study. These specific composition batches were selected as they were able to simulate the nonlinear mechanical properties of all types of human skin tested by Annaidh and colleagues [46,47]. Each composition was prepared by precisely measuring the four parts of the elastomer using experimental measuring cups and an Ohaus Adventurer Pro precision weight measuring device (Ohaus Corporation, Parsippany, NJ, USA), followed by thorough mixing for 1 min and curing for approximately 6.5 h. A universal testing machine (MTS Criterion Model 42, Eden Prairie, MN, USA) was used to conduct the uniaxial tests. Out of all the 23 compositions studied, one composition which simulated the nonlinear lower bound of human skin [46,47] was selected to further study the similarities in linear mechanical properties (low and high stretch moduli, and ultimate tensile stress) of the skin surrogate and the natural skin. This composition was named “90-10”, meaning 90 wt % of the two-part elastomer with a hardness of 30 A (45 wt % of part A and 45 wt % of part B), and 10 wt % of the two-part elastomer with a hardness of 00-10 (5 wt % of part A and 5 wt % of part B).

### 2.2. Mechanical Testing of Human Skin Tissue Surrogates

**Figure 3:**
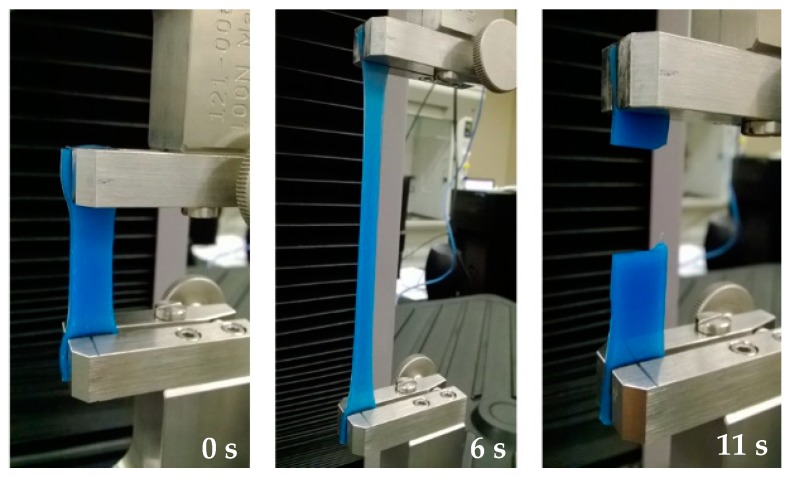
Uniaxial test on a skin surrogate specimen captured at the beginning and during the test.

A uniaxial test is the most common mechanical test performed on a universal testing machine to measure the load versus deformation behavior of any test specimen. However, several considerations need to be taken into account while testing soft materials, such as elastomers and the skin [51]. First, soft materials slip very easily, and thereby special grips coated with any material, which provides high friction against slippage, needs to be used. Second, strain rates significantly affect the load response of soft materials [52] and this phenomenon has been observed with the skin [47], which necessitates the use of a specific strain rate, so that results can be precisely compared with those from the literature. Third, the shape and size of the specimen may affect the test results [53]. All these issues were considered in our experimental framework. Special grips were used for specimen gripping, and a specific coupon size and a strain rate were used throughout the experiments. Additionally, a very small initial load (<0.1 N) was applied on each test coupon to ensure there was no slack to start with. A constant strain rate of 0.4 s^−1^ and a coupon size of 35 mm × 10 mm × 3 mm (after clamping) were selected for all the uniaxial tests on the soft surrogates, based on the uniaxial tests on human skin by Annaidh and colleagues [46,47]. For each of the tests, load (N) versus extension (mm) graphs were plotted. Figure 3 shows an ongoing uniaxial test until rupture of a skin surrogate coupon.

For post-processing of the raw load–extension curves obtained from the universal testing machine, a well-defined protocol was followed, comprising seven major steps: (i) any part of the plots, which showed negative load values, was trimmed off (which may arise from the specimen being slack initially); (ii) any part of the plots after the yield point was trimmed off, as they were insignificant for our analysis; (iii) the graphs were shifted (excluding the initial negative load values from positive displacements) to start from the origin; (iv) the engineering stress versus engineering strain plots were replotted as true stress versus true strain plots, which were obtained using Equations (1) and (2); (v) highly accurate fifth-degree polynomial trendlines were fit to each of the plots with coefficient of determination (*R*^2^) values between 0.95 and 1; (vi) all the plot strain (*x*-axis) steps and ranges were standardized (1 < *ε_true_* < 2 at a 0.01 increment step), and the respective stress values were calculated using the trendline equations obtained in step 5, and replotted; (vii) each stress–strain plot was converted to stress–stretch (*λ*) plots using Equation (3) as follows:

(1) $$\sigma_{true}=\sigma_{eng}\times (1+\varepsilon_{eng}),$$

(2) $$\varepsilon_{true}=\ln(1+\varepsilon_{eng}),$$

(3) λ=1+ε.

### 2.3. Nonlinear Material Modeling

Soft tissues are in general nonlinear materials, the properties of which are characterized using hyperelastic constitutive models, such as Fung, Mooney–Rivlin, Yeoh, Neo–Hookean, Ogden, Humphrey, Martins and Veronda–Westmann [44,51,54,55,56,57,58,59]. Hyperelastic material models are based on the definition of the strain energy function (denoted as *Ψ*), which depends on the type of material [60,61]. Any hyperelastic model is at least dependent on either the principal stretches (*λ*_1_, *λ*_2_ and *λ*_3_) along the three general Cartesian coordinate axes (*x*, *y* and *z*) or the Cauchy–Green tensor invariants (*I*_1_, *I*_2_ and *I*_3_, which are also functions of the principal stretches) [51], as shown in Equation (4). In this work, the Veronda–Westmann hyperelastic model was used to characterize the nonlinear mechanical behavior of the human skin surrogates, mainly because this model has been used previously in the literature to accurately characterize human skin [45,48,62,63]. The strain energy function of the Veronda–Westmann model is shown in Equation (8), with *c*_1_ and *c*_2_ denoted as the curve-fit material constants.

(4) $$\psi =\psi (I_{1},I_{2},I_{3}),$$

(5) $$I_{1}=\sum_{i=1}^{3}\lambda_{i}^{2},$$

(6) $$I_{2}=\sum_{i,j=1}^{3}\lambda_{i}^{2}\lambda_{j}^{2},$$

(7) $$I_{3}=\prod_{i=1}^{3}\lambda_{i}^{2},$$

(8) $$\psi_{Veronda-Westmann}=c_{1}[e^{c_{2}(I_{1}-3)}-1]-\frac{c_{1}c_{2}}{2}(I_{2}-3).$$

Uniaxial tests were conducted using the procedure outlined in the literature by Martins et al. [51], and the principal Cauchy stress was expressed in terms of the stretch and the strain energy function presented in Equation (9). Combined with the use of Equations (8) and (9), the nonlinear stress–strain behavior of the specimen was predicted using Equation (10) for uniaxial tests.

(9) $$\sigma_{1}=\lambda_{1}\frac{\partial \psi }{\partial \lambda_{1}}-\lambda_{3}\frac{\partial \psi }{\partial \lambda_{3}},\sigma_{2}=\sigma_{3}=0,$$

(10) $$\sigma_{Veronda-Westmann}=2(\lambda^{2}-\frac{1}{\lambda })c_{1}c_{2}(e^{c_{2}(I_{1}-3)}-\frac{1}{2\lambda }).$$

True stress versus true stretch data obtained from uniaxial tests were substituted in Equation (10) and the values of the material constants (*c*_1_ and *c*_2_) were determined. To evaluate the accuracy of the hyperelastic model chosen in predicting the skin material behavior, a correlation value (*R*^2^) calculation was conducted, where *R*^2^ can have a value from 0 to 1, with 1 representing the best fit and 0 representing the worst fit.

## 3. Results and Discussion

### 3.1. Mechanical Test of Human Skin Tissue Surrogates

**Figure 4:**
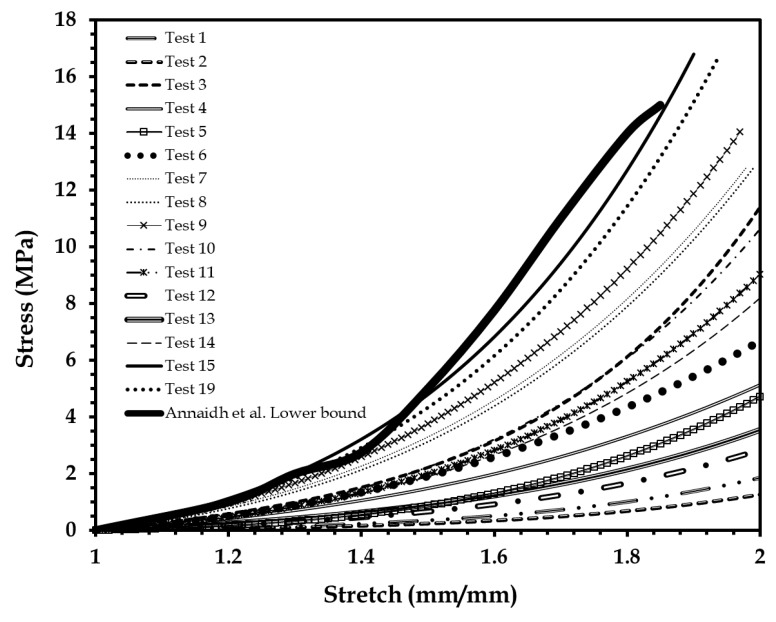
Stress–stretch plots of 16 elastomer specimens compared to human skin lower bound reported by Annaidh et al. [46]. Adapted from [46], Copyright 2012, with permissions from Elsevier.

The first batch of uniaxial mechanical tests were performed on elastomer-based skin surrogate coupons made by mixing different percentages of the four-part elastomer material, at a strain rate of 0.4 mm/s consistent with uniaxial tests on human skin [46]. Figure 4 shows 16 of the 23 test results (out of the 55 specimens tested), which are below the lower bound of the human skin mechanical properties identified by Annaidh et al. [46].

**Figure 5:**
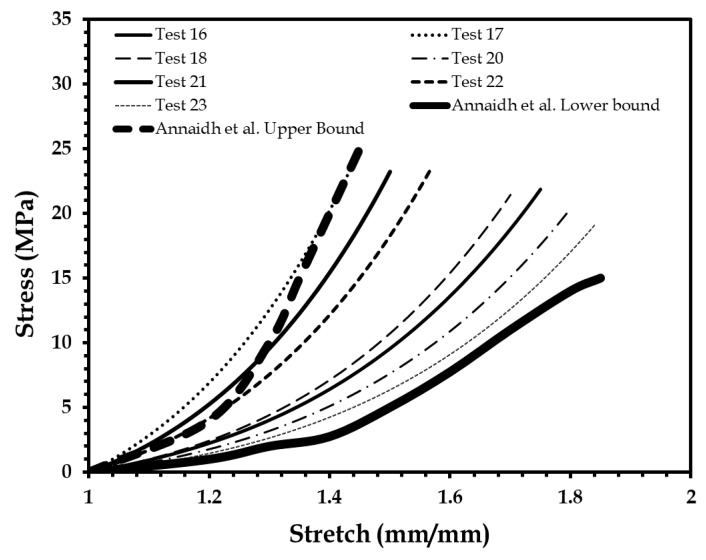
Stress–stretch plots of seven elastomer specimens tested to fabricate the human skin surrogate, compared to the lower and upper bounds of human skin test data reported by Annaidh et al. [46]. Adapted from [46], Copyright 2012, with permissions from Elsevier.

Figure 5 shows the nonlinear mechanical property of skin surrogates which lie within the human skin bounds [46]. The four-part mix ratio corresponding to these surrogates was found to be 46–57 wt % of part A and 37–47 wt % of part B of the two-part elastomer with a hardness of 30 A; and 3–5 wt % of part A and 3–5 wt % of part B of the two-part elastomer with a hardness of 00-10. 

### 3.2. Mechanical Modeling of Human Skin Tissue Surrogates at Different Body Sites

**Figure 6:**
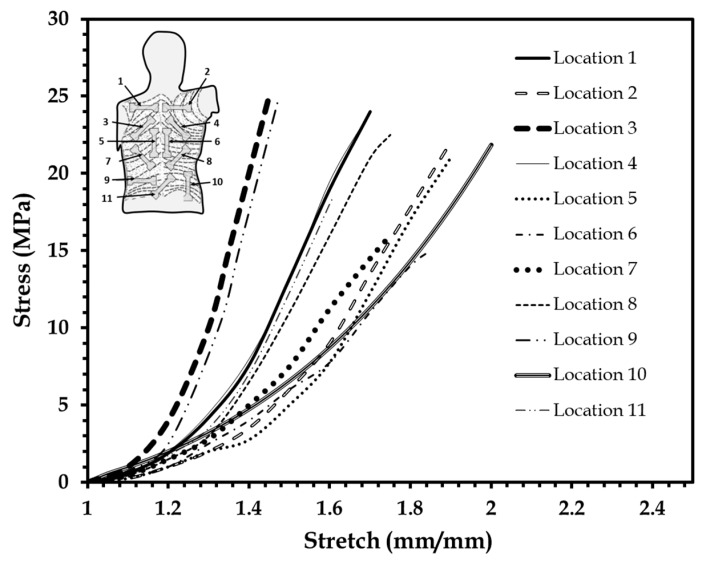
Stress–stretch plots of human skin test data. Reproduced from [46], Copyright 2012, with permissions from Elsevier.

Stress–stretch data for skin from different body locations were traced and replotted on Figure 6 from Annaidh and colleagues [46,47] for comparison with the stress–stretch plot of the skin surrogates shown in Figure 5. The body regions simulated were the upper, middle, and lower back, and the specimen orientations were at 0°, 45° and 90° with respect to the Langer lines as described in [46,47]. This comparison was similar to that attempted by Payne et al. [33,34]. Different elastomer compositions were identified, which closely simulated the human skin plot from a certain body location (Figure 6). Table 2 summarizes all these findings along with the average Veronda–Westmann hyperelastic material parameters estimated for the surrogates simulating human skin properties at different body locations. A good average *R*^2^ correlation index of 0.982 was estimated for these experiments and plots from [46].

### 3.3. Comparison of the Mechanical Properties of the 90-10 Skin Tissue Surrogate and Human Skin

**Figure 7:**
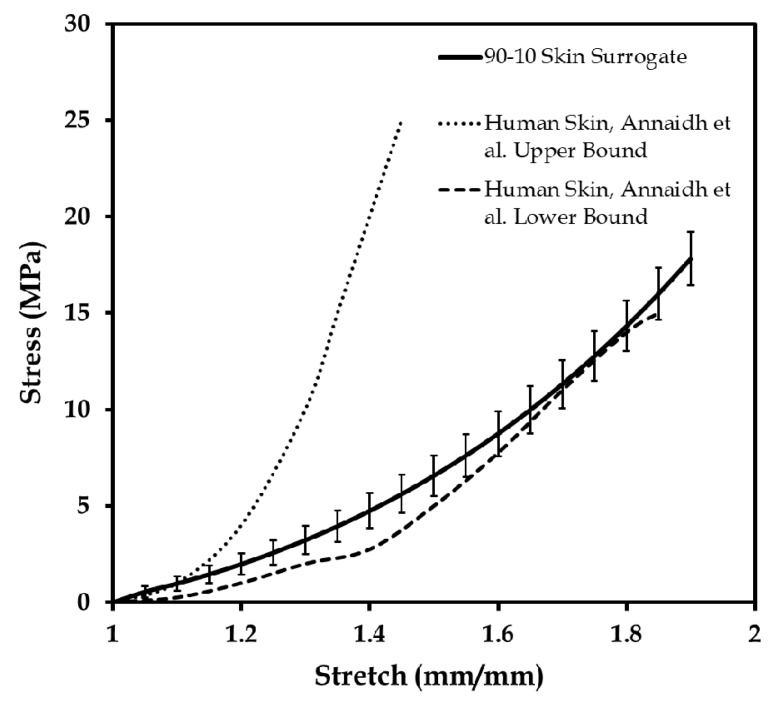
Stress–stretch plots of the 90-10 elastomer specimen under 36-time repeatability tests, compared to the lower and upper bounds of human skin test data reported by Annaidh et al. [46]. Adapted from [46], Copyright 2012, with permissions from Elsevier.

The 90-10 skin surrogate control specimen developed for simulating the lower bound of the human skin was studied with 36 uniaxial tests. Figure 7 shows the results of the repeatability tests together with the upper and lower bounds of human skin tested by Annaidh and colleagues [46,47]. The elastic modulus (at low and high stretch) and ultimate tensile stress of the 90-10 specimen were compared with the most comprehensive literature on mechanical properties of the human skin [33,34,46,64], described in Annaidh et al. [46]. Table 3 presents the results of the elastic moduli at low and high stretch, and ultimate tensile stress for the 36 “90-10” specimens tested for repeatability. Both the elastic modulus and ultimate stress values were found to be in the range of the human skin [46]. It should be further noted that the 90-10 surrogate specimen did not fracture like the natural human skin, and only compared well with the ultimate tensile stress values of the human skin [46].

## 4. Conclusions and Future Perspectives

In this work, the development and testing of novel human skin tissue surrogates were presented. The fabrication methodology, experimental framework, and hyperelastic characterization model were discussed in detail. Literature-based tests on natural human skin were employed as the basis of the experimental model presented here. Skin surrogates were developed with different four-part compositions to lie below and within the range of mechanical properties of human skin tested in [46]. In addition, surrogates were developed to simulate the mechanical properties of skin from different sites on the human body with high correlation (*R*^2^ of 0.97). Hyperelastic characterization parameters were estimated numerically for these specific skin surrogate compositions at different body sites. Additionally, a control skin surrogate specimen referred to as “90-10” was developed and used to conduct repeatability tests, and to compare with the elastic modulus at high and low stretch, and ultimate tensile stress of natural human skin. The surrogates developed in this work lay the foundation steps for biomechanical modeling of biofidelic human skin. Some of the potential biomechanical testing applications of these surrogates include the study of skin injury, the protective effect of personal protective equipment (PPE) and safety systems in sports (such as protective armors and sport gears), automotive industry, and the military. Additionally, in the prosthetics and cosmetics industries, these skin surrogates may be useful for studying the load response of skin implants, with the possibility of developing lighter and reliable substitutes.

The currently developed material system for human skin surrogates has several limitations, which should be acknowledged. First, an isotropic material was used to develop the skin surrogates. In reality, skin is anisotropic based on its location on the body, due to the presence of Langer or fiber lines. Second, only uniaxial tensile tests were employed in the current experimental framework because of the assumption of an isotropic material system. Third, in this work, a single and low strain rate was used to characterize the skin surrogates. In reality, varying strain rates can significantly change the mechanical response of skin, such as in the case of a blast, ballistic impact, or high-speed vehicular impact. Fourth, the tear and puncture performance of the isotropic skin surrogates are expected to be significantly different from that of the anisotropic natural skin, which may have implications on ballistic testing and surgical simulations. Fifth, cyclic or fatigue loading may lead to change in mechanical properties of natural tissue at high strains, which are not possible to predict with the testing of the highly elastic and isotropic skin surrogates. Sixth, natural skin pre-stress and load responses have been observed to vary with temperature, humidity, and ageing, neither of which can be simulated with the current elastomer-based skin surrogates. Seventh, the multi-layered structure of natural skin is connected to the underlying muscle, fat and other connective tissues contributing significantly to its mechanical properties. For the skin surrogates developed in this work, only a single dermal layer of the skin was modeled, which may have different biomechanical properties compared to the natural skin. In the future, composite-based anisotropic tissue surrogates need to be developed, which can simulate the mechanical properties of the multi-layered skin tissue. Such sophisticated surrogates will have to be tested with multiaxial dynamic and cyclic loads, and at varying stain rates to understand their damage and puncture properties. Simulating the effect of temperature, humidity, or ageing on the natural porous skin tissue, using the current elastomeric materials (used to develop the skin surrogates in this work), may be challenging due to its synthetic and nonporous nature. Structural modifications induced with specialized additive manufacturing techniques may be useful to bridge this gap. Ultimately, for realistic simulation of the load response of skin at different body sites, whole body surrogate models, such as a prosthetic limb made of skin and underlying tissue surrogates, will be necessary.

## Figures and Tables

**Table 1:** Four-part elastomer combinations tested for fabricating the human skin surrogates.

Test Specimen No.	Shore 00-10		Shore 30 A	
	Part A	Part B	Part A	Part B
1	45	45	5	5
2	45	45	5	5
3	15	15	35	35
4	42	42	8	8
5	42	42	8	8
6	35	35	15	15
7	12.5	12.5	37.5	37.5
8	12.5	12.5	37.5	37.5
9	10	10	40	40
10	15	15	35	35
11	15	15	35	35
12	45	45	5	5
13	42	42	8	8
14	25	25	25	25
15	5	5	45	45
16	3	3	47	47
17	3	3	57	37
18	3	3	47	47
19	5	5	45	45
20	5	5	47	43
21	3	3	54	40
22	3	3	52	42
23	4	4	46	46

**Table 2:** Skin surrogate compositions simulating human skin from different body locations presented in Figure 6, and the corresponding average hyperelastic model parameters.

Sample Location	Shore 00-10	Shore 30 A	Veronda–Westmann Hyperelastic Model Coefficients	
	Part A–Part B	Part A–Part B	*c*_1_	*c*_2_
1	3:7	51:39	27	0.29
2	8:2	44:46	16	0.26
3	7:3	61:29	35	0.33
4	4:6	52:38	28	0.3
5	7:3	45:45	15	0.23
6	5:5	45:45	13.1	0.21
7	2:8	48:42	18	0.20
8	5:5	47:43	22	0.26
9	6:4	58:32	33	0.30
10	8:2	42:48	14.4	0.27
11	7:3	48:42	24	0.28

**Table 3:** Comparison of mechanical properties of human skin and the 90–10 skin surrogate.

Test Specimen	Elastic Modulus (*E*) (Low Stretch Ratio)	Elastic Modulus (*E*) (High Stretch Ratio)	Ultimate Tensile Stress (MPa)
90-10 Human Skin Surrogate	2.163 ± 0.196	29.411 ± 2.261	16.23 ± 1.15
Human Skin ^1^	1.18 ± 0.88	83.3 ± 34.9	21.6 ± 8.4

^1^ Data reproduced from [46], Copyright 2012, with permissions from Elsevier.
